# Metagenomic Profiling of Diarrheagenic *Escherichia coli* Pathotypes Reveals Predominance of Diffusely Adherent and Enteroaggregative Subtypes and Mixed Signatures Among Under-Fives in Tanzania

**DOI:** 10.3390/cimb48080794

**Published:** 2026-08-05

**Authors:** Davis John Kuchaka, Mariana J. Shayo, Melkiory Beti, Patrick Kimu, Boaz D. Wadugu, Ignass P. Ignass, Gerald Phares, Masoud A. Juma, Happiness H. Kumburu, SeqTZ Consortium, Paul E. Kazyoba, Philip T. L. C. Clausen, Blandina T. Mmbaga, Emmanuel A. Mpolya, Tolbert B. Sonda

**Affiliations:** 1 School of Life Sciences and BioEngineering, The Nelson Mandela African Institution of Science and Technology, Arusha 23311, Tanzania; emmanuel.mpolya@nm-aist.ac.tz; 2 Kilimanjaro Clinical Research Institute, Moshi 25116, Tanzania; mariana.shayo@muhas.ac.tz (M.J.S.); m.beti@kcri.ac.tz (M.B.); p.kimu@kcri.ac.tz (P.K.); boazwadugu@gmail.com (B.D.W.); i.ignas@kcri.ac.tz (I.P.I.); gphares28@gmail.com (G.P.); sallass2012@gmail.com (M.A.J.); h.kumburu@kcri.ac.tz (H.H.K.); e.kussaga@kcri.ac.tz (S.C.); b.mmbaga@kcri.ac.tz (B.T.M.); 3 Department of Biomedical Science, School of Public Health, KCMC University, Moshi 25116, Tanzania; 4 Department of Paediatric and Child Health, School of Medicine, KCMC University, Moshi 25116, Tanzania; 5 Department of Biological and Pre-Clinical Studies, Muhimbili University of Health and Allied Sciences, Dar es Salaam 11102, Tanzania; 6 Government Chemist Laboratory Authority, Dar es Salaam 11101, Tanzania; 7 National Institute for Medical Research, Dar es Salaam 11101, Tanzania; paul.kazyoba@nimr.or.tz; 8 Department of Microbiology and Parasitology, State University of Zanzibar, Zanzibar 72208, Tanzania; 9 National Food Institute, Technical University of Denmark, 2800 Kongens Lyngby, Denmark; plan@food.dtu.dk; 10 Kilimanjaro Christian Medical Centre, Moshi 25116, Tanzania

**Keywords:** diarrheagenic *E. coli*, DAEC, EAEC, EPEC, ETEC, EIEC, nanopore metagenomics, GPMB, Tanzania, under-five diarrhoea, mixed virulence signatures, KMA

## Abstract

Diarrheagenic *Escherichia coli* (DEC) is a leading enteric pathogen in children under five in sub-Saharan Africa, yet conventional targeted assays fail to capture mixed virulence signatures or provide quantitative context relative to *E. coli* carriage. We applied a virulence-aware nanopore metagenomics workflow to characterise DEC pathotype distribution, mixed signatures, and virulence gene burden in 126 under-five stool metagenomes from six Tanzanian regions. Virulence support was quantified as aligned bases normalised to per-sample *E. coli*-aligned bases, termed GPMB (gene bases per million *E. coli*-aligned bases). At least one DEC virulence family was detected in 79/126 (62.7%) samples. *afa*/*dra* (diffusely adherent *E. coli* [DAEC] marker) was the most prevalent family (46/126, 36.5%); DAEC-containing pathotypes accounted for 46/79 (58.2%) of all assigned calls across all six regions. Mixed signatures occurred in 21/79 (26.6%) assigned samples, most commonly DAEC + enteroaggregative *E. coli* (EAEC) (*n* = 11). Enterotoxigenic *E. coli* (ETEC) was geographically concentrated in Mwanza. Age-stratified analysis revealed declining DAEC prevalence with age. Multivariable logistic regression found no significant independent associations between DEC positivity and age, sex, or rainfall. Unassigned samples had 6.8-fold lower *E. coli* carriage depth, implicating sequencing depth rather than pathotype absence as the primary non-detection driver. DAEC and EAEC should be elevated as priority DEC surveillance targets in Tanzania.

## 1. Introduction

Diarrhoeal disease remains a leading cause of morbidity and mortality in children under five years of age, responsible for approximately 500,000 deaths of children under five annually, with the greatest burden concentrated in sub-Saharan Africa and South Asia [1,2]. Among bacterial enteropathogens, diarrheagenic *Escherichia coli* (DEC) constitutes a heterogeneous group of pathotypes defined by distinct virulence gene repertoires. Six major DEC pathotypes are recognised: enteropathogenic (EPEC), enteroaggregative (EAEC), enterotoxigenic (ETEC), enteroinvasive (EIEC)/Shigella-like, diffusely adherent (DAEC), and Shiga toxin-producing (STEC) *E. coli* [1,3]. A recent African-wide systematic review reported pooled DAEC prevalences of up to 36% in paediatric diarrhoeal cohorts, underscoring the relevance of all major pathotypes [4].

EPEC is defined by the locus of enterocyte effacement (LEE) pathogenicity island, encoding a type III secretion system and the adhesin intimin (*eae*), causing attaching-and-effacing (A/E) lesions. Bundle-forming pili (*bfpA*) distinguish typical EPEC (tEPEC) from atypical EPEC (aEPEC) [5,6]. EAEC is characterised by aggregative adherence mediated by aggregative adherence fimbriae (AAF), with key markers *aggR*, *aap*, and *aaiC*; it is the leading cause of persistent diarrhoea and growth faltering in low- and middle-income countries (LMICs) [7]. ETEC produces heat-labile (LT; *elt*) and/or heat-stable (ST; *est*) enterotoxins and is a major target of vaccine development [2]. EIEC and *Shigella* share an invasion plasmid encoding *ipaH* and cause dysenteric illness [3,8]. DAEC is mediated by *afa*/*dra* adhesin operons and the *aatA* gene, disrupting brush border integrity, and has been under-studied relative to its clinical burden [9].

DEC transmission occurs principally via the faecal–oral route, driven by contaminated food and water, unsafe food handling and cross-contamination during preparation, and inadequate hygiene and sanitation infrastructure—risks amplified in young children by frequent hand-to-mouth behaviour and close household contact [1,2,3]. STEC in particular is classically linked to zoonotic and food-borne transmission via cattle and other ruminants, contaminated water, and person-to-person spread, with poor personal hygiene after animal contact identified as a specific risk factor [10]. Despite this recognised burden, STEC remains comparatively under-reported in sub-Saharan Africa; this has been attributed less to a lower true prevalence than to limited laboratory capacity for Shiga toxin-specific diagnostics in many African settings, likely resulting in under-ascertainment relative to high-income countries [10]. STEC classically presents with bloody diarrhoea and, in its most severe form, haemolytic uraemic syndrome, distinguishing it clinically from the predominantly watery, non-bloody presentations more typical of EAEC, ETEC, and DAEC.

A fundamental challenge in DEC surveillance is that virulence determinants are mobile genetic elements that can co-occur across or within strains, generating mixed signatures invisible to single-pathotype assays [3,11]. Conventional targeted methods report one pathotype per specimen, potentially missing co-occurring modules [12]. In Nigeria, whole-genome sequencing revealed hybrid DEC pathotypes in 65.8% of diarrhoeal cases [5].

Tanzania presents a compelling multi-region setting. Prior studies have documented DEC among under-five diarrhoeal presentations [13,14,15], but most data derive from single-site, culture-based, or targeted polymerase chain reaction (PCR) studies. Shotgun metagenomics simultaneously detects all virulence families within a specimen without pre-specifying targets, and nanopore long-read sequencing is increasingly deployable in resource-limited settings [16]. We quantify virulence support as aligned bases normalised to per-sample *E. coli*-aligned bases—GPMB (gene bases per million *E. coli*-aligned bases)—a read-length-independent metric accounting for differences in *E. coli* carriage.

This study aimed to: (i) characterise DEC virulence family prevalence and rule-based pathotype calls across six Tanzanian regions; (ii) quantify virulence burden using GPMB; (iii) describe mixed DEC signatures; (iv) examine the relationship between DEC positivity and individual-level covariates (age, sex, rainfall, temperature); and (v) identify drivers of the high unassigned fraction.

## 2. Materials and Methods

### 2.1. Study Design and Sample Collection

Stool specimens were collected from children under five years of age presenting with acute diarrhoea at public health facilities across six Tanzanian regions between May 2023 and April 2024. Collection sites represented multiple levels of the Tanzanian public health facility hierarchy: Mwanza (three facilities—Nyamagana District Hospital [*n* = 26], Buzuruga Health Centre [*n* = 12], and Sekou-Toure Regional Referral Hospital [*n* = 2]); Dodoma (Dodoma Regional Referral Hospital [*n* = 28]); Tabora (Kitete Regional Referral Hospital [*n* = 24]); Tanga (Magunga District Hospital, Korogwe District [*n* = 20]); Mbeya (Mbeya Regional Referral Hospital [*n* = 12]); and Zanzibar (Fuoni Primary Health Care Unit, West B District, Unguja [*n* = 3]). This multi-level design spanning primary health care units, district hospitals, and regional referral hospitals captures diarrhoeal presentations across a range of care-seeking contexts and clinical severity profiles. Specimens were stored at −80 °C prior to DNA extraction. The region-tagged analytic set comprised 126 metagenomes with complete metadata and quality-passing KMA outputs. One additional specimen produced quality-passing KMA output but could not be linked to a participant-level metadata record and was therefore excluded from the analytic set.

### 2.2. Ethical Approval

The study was conducted in accordance with the Declaration of Helsinki and approved by the National Institute for Medical Research (NIMR), Tanzania (protocol code NIMR/HQ/R.8c/Vol.I/2876). Informed consent was obtained from all caregivers of participating children.

### 2.3. DNA Extraction, Library Preparation, and Sequencing

DNA was extracted using the ZymoBIOMICS™ DNA/RNA Miniprep Kit (Zymo Research, Sunnyvale, CA, USA). Sequencing libraries were prepared using the Rapid Barcoding Kit 24 V14 (SQK-RBK114.24; Oxford Nanopore Technologies, Oxford, UK) and sequenced on MinION Mk1B with R10.4.1 flow cells. Basecalling used Dorado with the super-accuracy (SUP) model [17].

### 2.4. Two-Pass KMA Mapping and GPMB Quantification

Reads were mapped using KMA [18,19] in two sequential passes: (i) a genomic/species pass to derive per-sample *E. coli*-aligned base counts (ecoli_bp), and (ii) a virulence pass against a curated *E. coli* virulence panel with target-level filters (template identity ≥ 0.87; breadth ≥ 0.70). GPMB was computed as 10^6^ × vf_bp/ecoli_bp, where vf_bp is the sum of aligned bases across all quality-passing targets within a virulence family. GPMB quantifies relative sequence-level virulence gene representation in the metagenome and is not equivalent to viable pathogen load, strain abundance, or toxin expression; it should be interpreted as a measure of genomic virulence-determinant burden rather than a direct indicator of biological activity or disease severity. The full pipeline was implemented as a Snakemake workflow.

### 2.5. Positivity Criteria and Pathotype Assignment

A virulence family was considered present when vf_bp ≥ 3000 AND GPMB ≥ 5. Pathotypes were assigned by predefined marker rules: DAEC on *afa*/*dra* and/or *aatA*; EAEC on ≥1 of *aggR*, *aap*, *aaiC*; typical EPEC (tEPEC) on *bfpA* + *eae*; atypical EPEC (aEPEC) on *eae* alone; ETEC on *elt* and/or *est*; EIEC/Shigella-like on *ipaH*. Samples meeting criteria for ≥2 pathotypes received combined labels. Unassigned samples met no complete rule. Shiga toxin-producing *E. coli* (STEC) was assessed via *stx1* and *stx2* gene-family targets (137 allelic variants spanning *stx1a*/*c*/*d*/*e* and *stx2a*–*g*) within the same curated virulence panel; STEC positivity would have been assigned when *stx1* and/or *stx2* met the same positivity criteria (vf_bp ≥ 3000 AND GPMB ≥ 5) applied to the other pathotypes.

### 2.6. Covariate Data Collection and Linkage

Individual-level covariates were retrieved for all 126 analytic samples: age at diarrhoea onset (months), sex (male/female), and daily rainfall and mean daily temperature on the date of diarrhoea onset, obtained from the nearest regional meteorological station records (not facility-level measurements or interpolated climate products). The 102/126 samples with complete data for age, sex, and rainfall formed the complete-case regression set.

### 2.7. Statistical Analysis

Pathotype prevalence and GPMB distributions were summarised by region. GPMB values among positives are right-skewed; medians with interquartile ranges (IQRs) and geometric means are reported. Regional comparisons were adjusted using the Benjamini–Hochberg (BH) false discovery rate (FDR) procedure [20]; significance was defined at FDR ≤ 0.05. Age-stratified pathotype prevalence was tabulated across five strata (<6, 6–11, 12–23, 24–35, 36–59 months). Multivariable logistic regression was fitted for DEC+ (any), DAEC, and EAEC outcomes (*n* = 102 complete cases) with standardised age, rainfall, and sex as predictors. Events per variable (EPV) were DEC+ 21.7 (adequate), DAEC 12.3 (acceptable), and EAEC 5.3 (underpowered; interpret with caution). ETEC and EIEC were not modelled due to insufficient events. Samples were nested within eight collection facilities across six regions, with several facilities contributing fewer than five samples (e.g., Sekou-Toure Regional Referral Hospital, *n* = 2; Fuoni Primary Health Care Unit, *n* = 3). This precluded reliable estimation of facility-level random effects or cluster-robust standard errors, both of which require substantially more clusters than are available here; multivariable regression results are therefore presented as descriptive/exploratory characterisations of covariate–outcome associations rather than confirmatory inference accounting for facility-level clustering. All analyses were performed in Python version 3.12.3 (pandas v3.0.2, scipy v1.17.1, statsmodels v0.14.6).

## 3. Results

### 3.1. Analytic Set and Sequencing Characteristics

The analytic set comprised 126 stool metagenomes across six regions (Table 1): Mwanza (*n* = 40), Dodoma (*n* = 28), Tabora (*n* = 23), Tanga (*n* = 20), Mbeya (*n* = 12), and Zanzibar (*n* = 3). Collection spanned May 2023–April 2024, covering both rainy and dry seasons across the study regions. Total read counts ranged from 2890 to 2,137,305 (median 267,734). *E. coli*-aligned bases ranged over >3000-fold (732,743 to 2,530,450,975; median 41.8 Mbp). The human read fraction ranged from 0 to 98%, and 23 samples (18.1%) exceeded 50% human reads.

### 3.2. Covariate Data Quality

Of 126 matched samples, age was available for 102 (81.0%; median 12.1 months, IQR 8.3–19.3; range 1.8–46.4 months); sex was available for 107 (84.9%; 58.3% male). Rainfall was available for 119 (94.4%) but was severely zero-inflated: 73.1% recorded 0 mm; meaningful variation was limited to Mwanza (22/40 non-zero) and Tanga (8/20 non-zero). Mbeya, Tabora, and Zanzibar were all-zero. The median temperature was 23.6 °C (range 20.2–28.9 °C).

### 3.3. E. coli Carriage Depth and the Unassigned Fraction

Of 126 samples, 47 (37.3%) were unassigned (DEC panel neg). Unassigned samples had markedly lower *E. coli*-aligned bases than assigned samples (median 9.9 Mbp [IQR 3.2–27.7] vs. 67.1 Mbp [IQR 32.9–158.7]; 6.8-fold difference; *p* < 0.001, Mann–Whitney U). This pattern was consistent across all six regions.

### 3.4. DEC Virulence Family Prevalence

**Figure 1:**
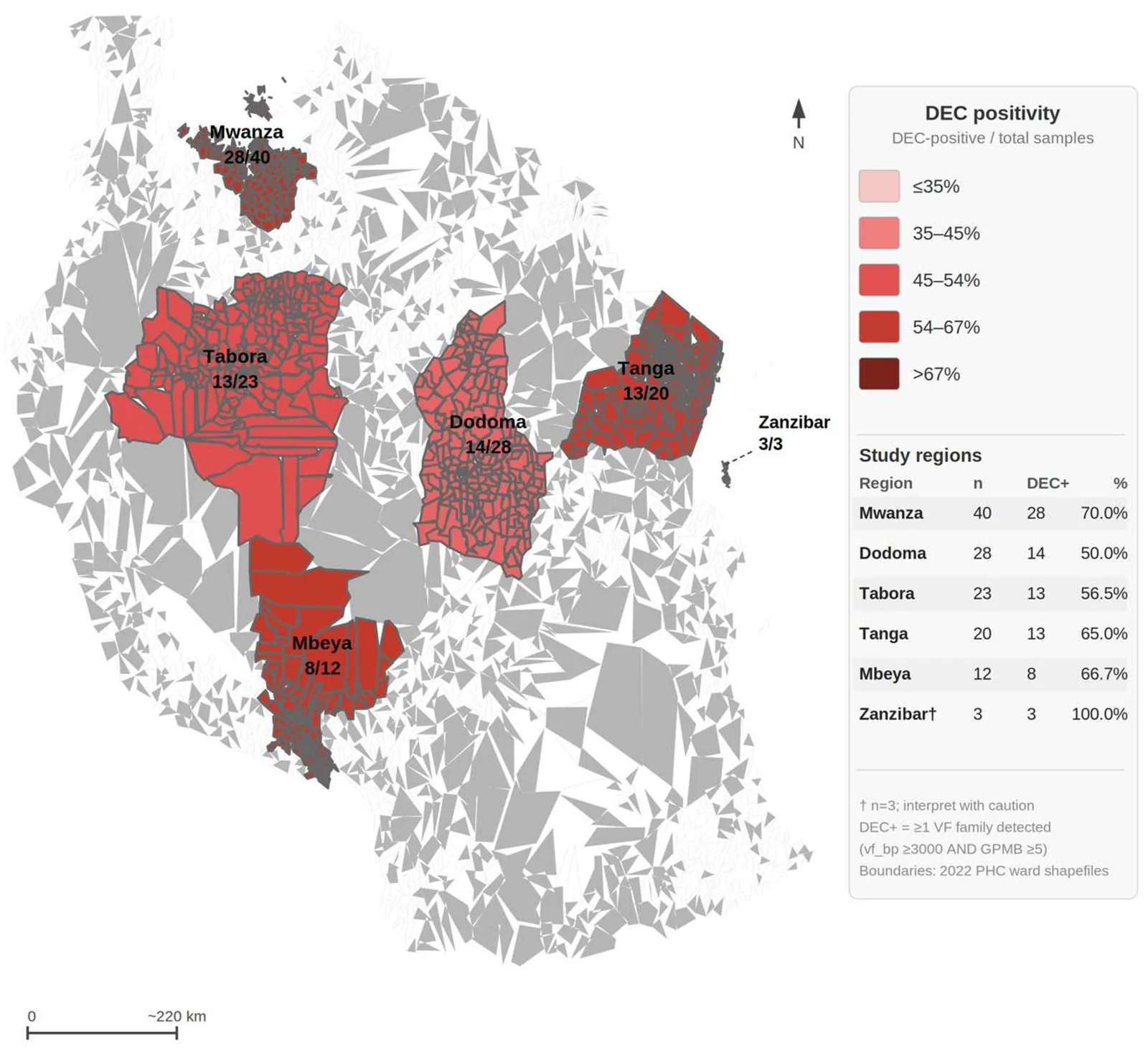
Choropleth map of DEC positivity across six study regions in Tanzania. Colour encodes the proportion of DEC-positive samples (red gradient; darker = higher positivity). Labels show DEC-positive count/total (e.g., 28/40). Region boundaries derived from the Tanzania 2022 Population and Housing Census (PHC) ward-level shapefiles. Zanzibar = Mjini Magharibi (Zanzibar West main island) only; *n* = 3; interpret with caution. Non-study regions shown in grey. DEC+, ≥1 DEC virulence family meeting positivity criteria.

At least one DEC virulence family met positivity criteria in 79/126 (62.7%) samples. *afa*/*dra* (DAEC marker) was the most prevalent family (46/126, 36.5%), followed by *aatA* (22.2%), *aggR* (EAEC; 15.9%), *aap* (EAEC; 15.1%), *eae* (EPEC/aEPEC; 15.1%), *bfpA* (10.3%), *aaiC* (EAEC; 7.9%), *elt* (ETEC LT; 7.1%), *ipaH* (EIEC/Shigella-like; 6.3%), and *est* (ETEC ST; 1.6%). No sample met positivity criteria for any *stx1* or *stx2* allelic variant; STEC was not detected in this cohort (0/126). Regional prevalence is shown in Table 2 and Figure 1.

### 3.5. Pathotype Distribution and Regional Heterogeneity

**Figure 2:**
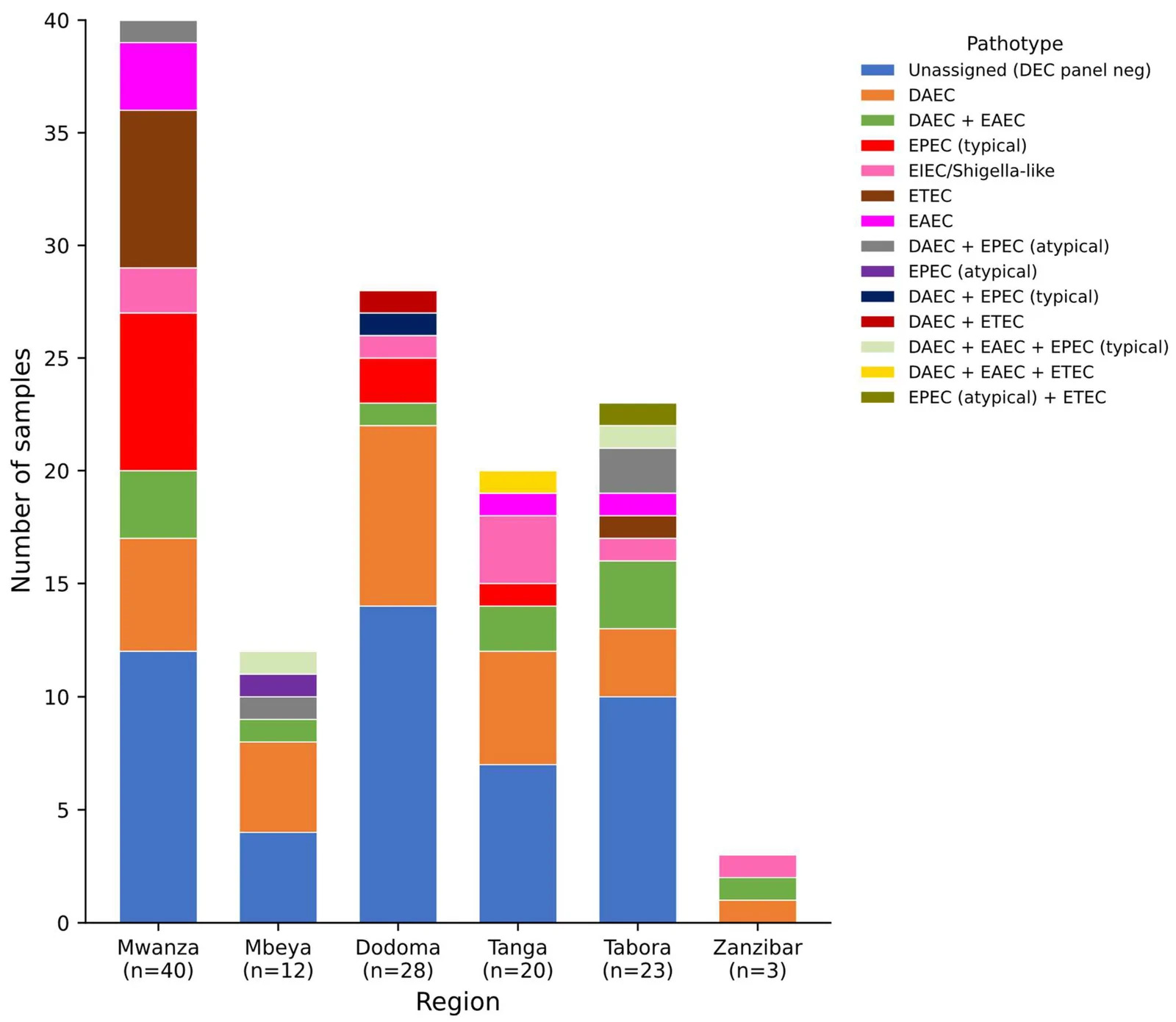
Regional distribution of DEC pathotype calls across 126 under-five stool metagenomes from Tanzania. Stacked bar chart showing pathotype assignments per region. Unassigned (DEC panel neg) = blue. Mixed labels indicate ≥2 co-assigned pathotypes. Positivity defined as vf_bp ≥ 3000 AND GPMB ≥ 5, with target-level identity ≥ 0.87 and breadth ≥ 0.70. Abbreviations: DAEC, diffusely adherent *E. coli*; EAEC, enteroaggregative *E. coli*; EPEC, enteropathogenic *E. coli*; ETEC, enterotoxigenic *E. coli*; EIEC, enteroinvasive *E. coli*; GPMB, gene bases per million *E. coli*-aligned bases.

Among 79 assigned samples, DAEC alone was the most frequent call (*n* = 26, 32.9%), followed by DAEC + EAEC (*n* = 11, 13.9%), EPEC typical (*n* = 10, 12.7%), EIEC/Shigella-like (*n* = 8, 10.1%), ETEC (*n* = 8, 10.1%), EAEC alone (*n* = 5, 6.3%), and DAEC + EPEC atypical (*n* = 4, 5.1%). DAEC-containing pathotypes accounted for 46/79 (58.2%) of assigned calls across all six regions. Within Mwanza, facility-level disaggregation revealed striking heterogeneity: all seven ETEC calls and all seven typical EPEC calls from Mwanza originated exclusively from Nyamagana District Hospital (NYG; *n* = 26), with no ETEC or typical EPEC detected at Buzuruga Health Centre (BZG; *n* = 12) or Sekou-Toure Regional Referral Hospital (MWZ; *n* = 2). By contrast, all three Mwanza mixed-signature samples were from Buzuruga Health Centre. EIEC/Shigella-like calls were distributed across five regions, with the highest count in Tanga (*n* = 3, all from Magunga District Hospital, Korogwe District). A full regional breakdown is shown in Table 3 and Figure 2.

### 3.6. Mixed DEC Signatures

Mixed signatures were detected in 21/79 (26.6%) assigned samples. DAEC + EAEC was the dominant combination (*n* = 11, 52.4% of mixed calls), present in all five well-sampled regions. Other combinations included DAEC + EPEC atypical (*n* = 4), DAEC + EAEC + EPEC typical (*n* = 2), and four additional combinations (*n* = 1 each). Tabora had the highest mixed-signature rate among assigned samples (7/13, 53.8%); the median of *E. coli*-aligned bases among Tabora mixed-signature samples was 150 Mbp (range 58–743 Mbp).

### 3.7. GPMB: Virulence Burden Among Positive Samples

GPMB distributions were right-skewed for all families (Table 4). *ipaH* (EIEC/Shigella-like) showed the highest median GPMB (overall 2157 [IQR 1071–5039]); however, five of six highest-GPMB *ipaH* samples had human read fractions of 92–98% and *E. coli*-aligned bases of 1.3–7.7 Mbp, consistent with denominator-compression inflation. The two Mwanza *ipaH*-positive samples (human fraction 15%; ecoli_bp ≥ 71 Mbp) yielded GPMB of 207. For *aatA*, the median GPMB was highest in Mbeya (683.2 [IQR 351.8–942.5]) and Tabora (459.1 [IQR 256.3–557.8]). For *elt* (ETEC), the median GPMB in Mwanza was 475.4 (geometric mean 513).

### 3.8. Age-Stratified Pathotype Prevalence

**Figure 3:**
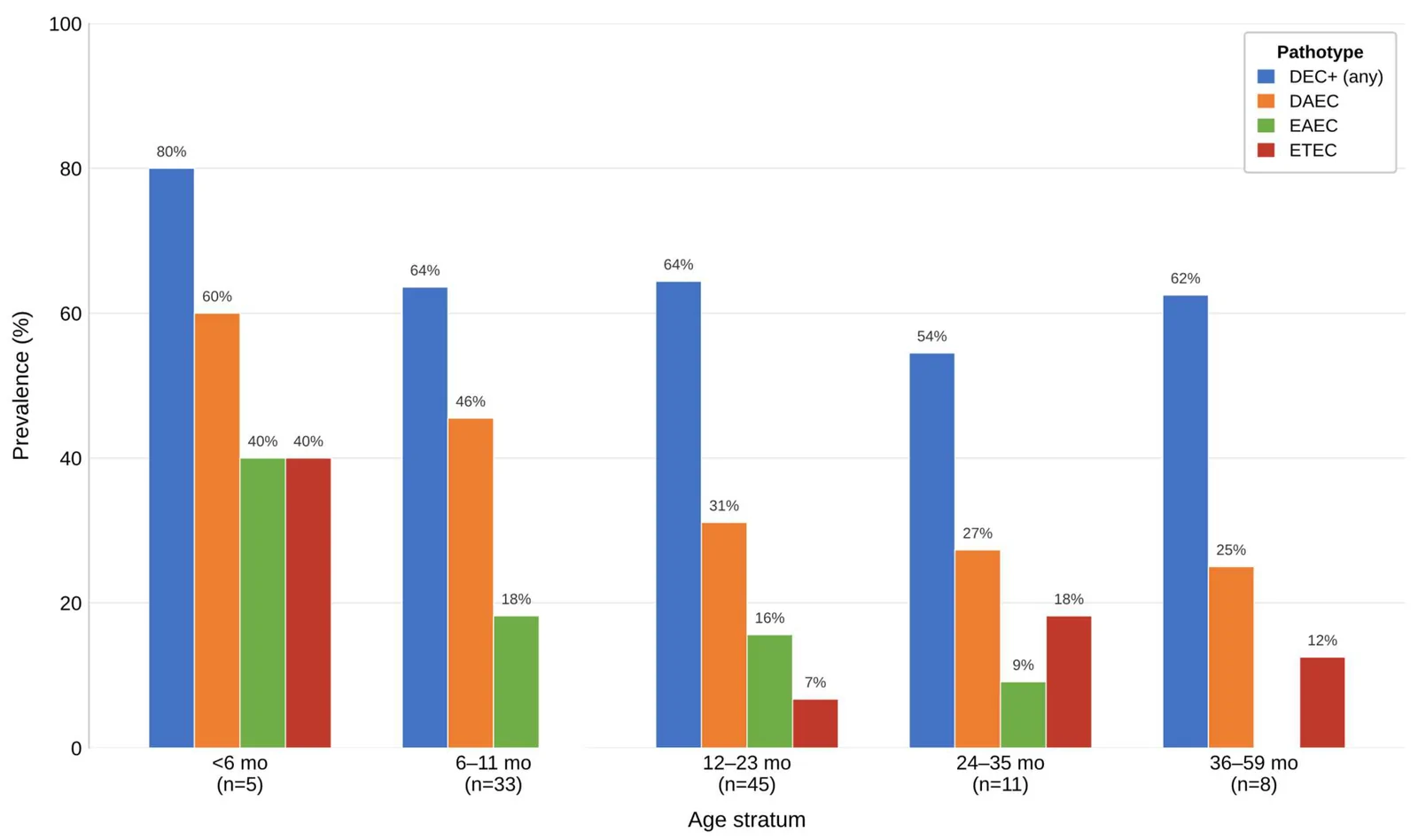
Age-stratified DEC pathotype prevalence across five under-five age strata. Grouped bar chart showing prevalence (%) of DEC+ (any), DAEC, EAEC, and ETEC across strata of <6, 6–11, 12–23, 24–35, and 36–59 months. *n* = 102 samples with age data. DAEC prevalence declines monotonically with age; EAEC is absent in the 36–59-month stratum. Strata with *n* < 10 (<6 months; 36–59 months) should be interpreted with caution.

Age data were available for 102/126 matched samples. Median age was 12.1 months (range 1.8–46.4). DAEC prevalence showed a declining pattern across strata: 60.0% (<6 months), 45.5% (6–11 months), 31.1% (12–23 months), 27.3% (24–35 months), and 25.0% (36–59 months). EAEC was absent in the 36–59-month stratum. ETEC appeared only in children aged ≥12 months (Table 5; Figure 3).

### 3.9. Multivariable Logistic Regression

For the 102 complete cases, multivariable logistic regression found no significant associations between DEC positivity and age (OR = 0.97, 95%CI 0.65–1.44, *p* = 0.87), rainfall (OR = 1.13, 95%CI 0.68–1.88, *p* = 0.65), or sex (OR = 0.73, 95%CI 0.32–1.69, *p* = 0.46). For DAEC, age showed a non-significant decreasing trend (OR = 0.74, 95%CI 0.47–1.15, *p* = 0.17). For EAEC, an inverse age trend approached significance (OR = 0.48, 95%CI 0.21–1.08, *p* = 0.07), but the model was underpowered (EPV = 5.3). Binary rainfall (any >0 mm vs. none) showed no association with DEC positivity (58.8% vs. 65.9%, *p* = 0.61). Temperature was not associated with DEC positivity (*p* > 0.99). Full regression results are shown in Table 6.

## 4. Discussion

This multi-region nanopore metagenomics study characterises DEC pathotype distribution in Tanzanian under-five diarrhoeal stools and integrates individual-level covariates to contextualise occurrence. Several findings carry epidemiological, clinical, and methodological significance.

### 4.1. DAEC Predominance Across All Regions and Ages

*afa*/*dra*-positive DAEC was detected in 36.5% of all 126 metagenomes and comprised 58.2% of assigned calls, present across all six regions and all age strata. The declining DAEC prevalence with age (60.0% to 25.0%) is consistent with the development of protective immunity to Afa/Dr adhesins and is corroborated by prior African cohort data [4,15]. It should be noted that, in multivariable regression, the association between age and DAEC positivity did not reach statistical significance (OR = 0.74, 95% CI 0.47–1.15, *p* = 0.17; Table 6), and the prevalence decline described above should therefore be interpreted as a descriptive pattern warranting confirmation in larger, adequately powered cohorts, rather than as an established epidemiological trend. These data nonetheless support elevating DAEC to a priority surveillance target in Tanzania’s national diarrhoeal disease framework.

### 4.2. EAEC and the Inverse Age Trend

The inverse association between age and EAEC detection (OR = 0.48, *p* = 0.07) is directionally consistent with EAEC being a predominant pathogen in younger children where immune control of aggregative adherence fimbriae is incomplete [7]. EAEC is the leading cause of persistent diarrhoea and growth faltering in LMICs [7], and its disappearance from the 36–59-month stratum suggests that the critical EAEC risk window may be the first three years of life. However, two strata carry important interpretive caveats: the <6-month stratum (*n* = 5) and the 36–59-month stratum (*n* = 8) are both below the threshold of *n* = 10 required for reliable prevalence estimation. The apparent 60.0% DAEC prevalence in infants under six months and the 0.0% EAEC rate in older toddlers should therefore be treated as directional signals rather than stable estimates. The most statistically reliable findings sit in the 6–11 month (*n* = 33) and 12–23 month (*n* = 45) strata, where the declining DAEC trend and the moderate EAEC prevalence are supported by adequate sample sizes. Larger, age-balanced cohorts are needed to confirm these patterns across the full under-five age spectrum.

### 4.3. Mixed Signatures: Biological Plausibility and Surveillance Implications

Mixed DEC signatures in 26.6% of assigned samples, dominated by DAEC + EAEC (52.4% of mixed calls), confirm that the multi-virulence-module landscape of DEC carriage cannot be captured by single-target assays. Co-occurrence of *afa*/*dra* with EAEC markers is biologically plausible: both sets of determinants are plasmid-encoded and can co-reside within a single *E. coli* strain [3]. The elevated mixed-signature rate at Kitete Regional Referral Hospital in Tabora (53.8% of assigned calls) was not explained by low *E. coli* depth. Kitete, established in 1906, serves as the regional referral facility for the large, sparsely populated Tabora region; its case mix likely includes children with more severe or prolonged diarrhoeal illness referred from peripheral facilities across a geographically dispersed catchment. Whether this case mix enrichment contributes to the higher observed mixed-signature rate, for example, by selecting for children with co-infections or complex DEC profiles, is a hypothesis warranting investigation in a study with individual-level clinical severity data.

### 4.4. ETEC and EPEC Concentration at Nyamagana District Hospital

All seven ETEC calls and all seven typical EPEC calls within Mwanza originated exclusively from Nyamagana District Hospital, with none detected at Buzuruga Health Centre or Sekou-Toure Regional Referral Hospital. This facility-level concentration, rather than a diffuse regional signal, has important interpretive implications. Nyamagana District Hospital serves an urban catchment in Nyamagana Municipality, and its case mix may differ from that of Buzuruga Health Centre in Ilemela District, a peri-urban setting with different WASH access profiles and population density. The co-concentration of both ETEC and tEPEC at a single district hospital may reflect a localised transmission cluster within the Nyamagana catchment, differential care-seeking for specific diarrhoeal presentations (ETEC-associated profuse watery diarrhoea may prompt earlier facility attendance), or referral patterns that enrich for more severe enteric illness. ETEC is a leading vaccine-preventable diarrhoeal pathogen with several LT- and ST-based candidates in clinical development [2]; these findings suggest that any ETEC vaccine trial siting assessment for Mwanza should investigate facility-level rather than region-level burden distributions.

### 4.5. EIEC/Shigella-like Signal and the Korogwe Setting

All three EIEC/Shigella-like calls from Tanga originated from Magunga District Hospital in Korogwe District, an inland riverine setting along the Pangani River valley, ecologically distinct from coastal Tanga Municipality. The Pangani basin environment, characterised by agricultural smallholder communities with surface water dependence, may present distinct EIEC/*Shigella* transmission risks compared to the coastal zone. This geographic specificity should temper any interpretation of the Tanga EIEC signal as representative of the entire Tanga region, and reinforces the importance of reporting collection facility locations alongside regional labels in multi-site metagenomics studies.

### 4.6. Rainfall and Covariate Null Results

No significant associations were found between continuous rainfall, age, sex, or temperature and DEC positivity. Rainfall was severely zero-inflated (73.1% zeros), with variation restricted to Mwanza and Tanga, rendering the continuous analysis near-equivalent to a binary variable. Notably, the 12-month collection window (May 2023–April 2024) spanned both rainy and dry seasons across multiple regions, reducing the likelihood that the overall DEC positivity findings are attributable to seasonal sampling bias; nevertheless, the zero-inflation of site-level rainfall records precluded a meaningful continuous rainfall analysis. This null result therefore reflects data structural limitations rather than a true absence of ecological associations; rainfall–DEC relationships should be revisited with temporally matched, region-representative meteorological data in future studies. The negative age result for DEC+ overall reflects the narrow under-five age window and DAEC dominance across all strata. Sex was confirmed as a non-confounder of regional comparisons.

### 4.7. GPMB as a Normalised Burden Metric

GPMB provides a read-length-independent, transparent framework for quantifying virulence evidence in nanopore metagenomes. GPMB quantifies relative sequence-level virulence gene representation in the metagenome and is not equivalent to viable pathogen load, strain abundance, or toxin expression; it should be interpreted as a measure of genomic virulence-determinant burden rather than a direct indicator of biological activity or disease severity. As demonstrated by the *ipaH* findings, GPMB is additionally vulnerable to denominator-compression inflation when human read fractions exceed ~90% and ecoli_bp falls below ~10 Mbp. We recommend reporting human read fraction and ecoli_bp as mandatory quality columns in metagenomics-based DEC studies.

### 4.8. Unassigned Fraction as a Depth Signal

The 6.8-fold lower median *E. coli*-aligned bases in unassigned samples strongly implicates insufficient *E. coli* read depth, not pathotype absence as the primary non-detection driver. Future studies should apply minimum ecoli_bp thresholds (suggested ≥10 Mbp) for inclusion in DEC prevalence analyses.

### 4.9. STEC Screening and Clinical Presentation

Despite comprehensive screening across 137 *stx1*/*stx2* allelic variants, STEC was not detected in any sample. The under-reporting of STEC in sub-Saharan Africa has previously been attributed more to limited laboratory capacity for Shiga toxin-specific diagnostics than to genuinely lower prevalence [10], making the sequence-based screening applied here—which did not rely on culture-based serotyping or toxin immunoassays—a methodological strength rather than a further source of under-ascertainment. Nonetheless, since STEC classically presents with bloody or dysenteric stool, a characteristic not recorded in this study, we cannot fully exclude case mix-driven under-ascertainment. Future studies capturing stool character alongside comprehensive sequence-based screening would help resolve whether STEC’s absence here reflects true local epidemiology or sampling effects.

## 5. Conclusions

Virulence-aware nanopore metagenomics establishes DAEC as the dominant DEC pathotype in Tanzanian under-five diarrhoea across six ecologically diverse regions, with mixed DAEC + EAEC signatures common across all settings. ETEC is geographically concentrated in Mwanza, with a high virulence burden and with implications for geographically targeted vaccine trial design. Age-stratified analysis reveals a declining DAEC prevalence and an inverse EAEC–age trend consistent with age-dependent susceptibility patterns. Sex was confirmed as a non-confounder; rainfall analysis was constrained by severe zero-inflation. The high unassigned fraction is primarily explained by low *E. coli* carriage depth rather than pathotype absence. GPMB-based normalisation provides a transparent, assumption-light framework for DEC metagenomics that requires explicit quality thresholds for human read fraction and minimum *E. coli*-aligned bases. These findings advance the epidemiological characterisation of DEC in East Africa and provide a quantitative, reproducible foundation for larger, clinically annotated metagenomics studies in the region.

## Figures and Tables

**Table 1:** Analytic set: sample counts, DEC positivity, and mixed-signature rates by region.

Region	Total (*n*)	DEC+ *n* (%)	Mixed *n* (%) *
Mwanza	40	28 (70.0)	4 (14.3)
Dodoma	28	14 (50.0)	3 (21.4)
Tabora	23	13 (56.5)	7 (53.8)
Tanga	20	13 (65.0)	3 (23.1)
Mbeya	12	8 (66.7)	3 (37.5)
Zanzibar	3	3 (100.0)	1 (33.3)
Total	126	79 (62.7)	21 (26.6)

* Mixed: proportion of DEC-assigned samples within each region with ≥2 pathotype designations. DEC+, any assigned DEC pathotype.

**Table 2:** DEC virulence family prevalence overall and by region. Values are *n* (%).

VF Family	Pathotype Marker	Total (*n* = 126)	Mwanza (*n* = 40)	Dodoma (*n* = 28)	Tanga (*n* = 20)	Tabora (*n* = 23)	Mbeya (*n* = 12)	Zanzibar (*n* = 3)
*afa*/*dra*	DAEC	46 (36.5)	9 (22.5)	11 (39.3)	8 (40.0)	9 (39.1)	7 (58.3)	2 (66.7)
*aatA*	DAEC	28 (22.2)	8 (20.0)	5 (17.9)	5 (25.0)	6 (26.1)	3 (25.0)	1 (33.3)
*aggR*	EAEC	20 (15.9)	6 (15.0)	2 (7.1)	4 (20.0)	5 (21.7)	2 (16.7)	1 (33.3)
*aap*	EAEC	19 (15.1)	6 (15.0)	1 (3.6)	2 (10.0)	5 (21.7)	4 (33.3)	1 (33.3)
*eae*	EPEC/aEPEC	19 (15.1)	8 (20.0)	3 (10.7)	1 (5.0)	4 (17.4)	3 (25.0)	0 (0.0)
*bfpA*	tEPEC	13 (10.3)	7 (17.5)	3 (10.7)	1 (5.0)	1 (4.3)	1 (8.3)	0 (0.0)
*aaiC*	EAEC	10 (7.9)	5 (12.5)	1 (3.6)	1 (5.0)	3 (13.0)	0 (0.0)	0 (0.0)
*elt*	ETEC (LT)	9 (7.1)	7 (17.5)	1 (3.6)	0 (0.0)	1 (4.3)	0 (0.0)	0 (0.0)
*ipaH*	EIEC/Shig.	8 (6.3)	2 (5.0)	1 (3.6)	3 (15.0)	1 (4.3)	0 (0.0)	1 (33.3)
*est*	ETEC (ST)	2 (1.6)	0 (0.0)	0 (0.0)	1 (5.0)	1 (4.3)	0 (0.0)	0 (0.0)

DAEC, diffusely adherent *E. coli*; EAEC, enteroaggregative *E. coli*; tEPEC, typical enteropathogenic *E. coli*; aEPEC, atypical EPEC; ETEC, enterotoxigenic *E. coli*; LT, heat-labile toxin; ST, heat-stable toxin; EIEC/Shig., EIEC/Shigella-like.

**Table 3:** Distribution of DEC pathotype calls by region.

Pathotype	Mwanza (*n* = 40)	Dodoma (*n* = 28)	Tabora (*n* = 23)	Tanga (*n* = 20)	Mbeya (*n* = 12)	Zanzibar (*n* = 3)	Total (*n* = 126)
Unassigned (DEC panel neg)	12	14	10	7	4	0	47
DAEC	5	8	3	5	4	1	26
DAEC + EAEC	3	1	3	2	1	1	11
EPEC (typical)	7	2	0	1	0	0	10
EIEC/Shigella-like	2	1	1	3	0	1	8
ETEC	7	0	1	0	0	0	8
EAEC	3	0	1	1	0	0	5
DAEC + EPEC (atypical)	1	0	2	0	1	0	4
Other mixed	0	2	1	2	0	0	5
Total assigned	28	14	13	13	8	3	79

‘Other mixed’ includes DAEC + ETEC (*n* = 1); DAEC + EAEC + EPEC typical (*n* = 2); DAEC + EAEC + ETEC (*n* = 1); EPEC atypical + ETEC (*n* = 1).

**Table 4:** Median GPMB [IQR] by virulence family and region (among positive samples). GPMB, gene bases per million *E. coli*-aligned bases.

VF Family	Mwanza (*n* pos)	Dodoma (*n* pos)	Tanga (*n* pos)	Tabora (*n* pos)	Mbeya (*n* pos)	Zanzibar (*n* pos)
*afa*/*dra*	116.8 [47.6–176.1]; *n* = 9	286.8 [77.0–497.0]; *n* = 11	215.1 [63.7–609.5]; *n* = 8	135.9 [51.1–180.7]; *n* = 9	320.0 [134.4–781.1]; *n* = 7	206.7; *n* = 2
*aatA*	179.6 [70.1–340.2]; *n* = 8	109.2 [72.3–234.3]; *n* = 5	173.4 [12.3–385.5]; *n* = 5	459.1 [256.3–557.8]; *n* = 6	683.2 [351.8–942.5]; *n* = 3	57.3; *n* = 1
*aggR*	165.9 [75.1–266.9]; *n* = 6	367.9 [40.3–695.5]; *n* = 2	109.1 [16.2–274.2]; *n* = 4	310.1 [252.2–320.8]; *n* = 5	246.2 [223.6–268.8]; *n* = 2	85.7; *n* = 1
*eae*	379.2 [282.8–570.4]; *n* = 8	348.8 [222.4–471.6]; *n* = 3	532.1; *n* = 1	42.0 [11.8–515.2]; *n* = 4	77.5 [15.8–546.9]; *n* = 3	—
*elt*	475.4 [355.4–766.0]; *n* = 7	210.1; *n* = 1	—	288.1; *n* = 1	—	—
*ipaH* †	206.7; *n* = 2	1070.5; *n* = 1	3467.7 [1965.3–5039.2]; *n* = 3	2349.1; *n* = 1	—	5138.4; *n* = 1

† Five of six highest-GPMB ipaH samples had human read fractions 92–98%; GPMB values may be inflated by low ecoli_bp denominators. —, no positive samples in that region. Values shown as median [IQR] where *n* ≥ 2; single values without IQR.

**Table 5:** Age-stratified DEC pathotype prevalence (*n* = 102 samples with age data).

Age Stratum	*n*	DEC+ *n* (%)	DAEC *n* (%)	EAEC *n* (%)	ETEC *n* (%)	EIEC *n* (%)	Mixed *n* (%)
<6 months	5	4 (80.0)	3 (60.0)	2 (40.0)	2 (40.0)	0 (0.0)	2 (40.0)
6–11 months	33	21 (63.6)	15 (45.5)	6 (18.2)	0 (0.0)	1 (3.0)	6 (18.2)
12–23 months	45	29 (64.4)	14 (31.1)	7 (15.6)	3 (6.7)	3 (6.7)	6 (13.3)
24–35 months	11	6 (54.5)	3 (27.3)	1 (9.1)	2 (18.2)	2 (18.2)	1 (9.1)
36–59 months	8	5 (62.5)	2 (25.0)	0 (0.0)	1 (12.5)	0 (0.0)	0 (0.0)
Total	102	65 (63.7)	37 (36.3)	16 (15.7)	8 (7.8)	6 (5.9)	15 (14.7)

Strata with *n* < 10 (<6 months; 36–59 months) should be interpreted with caution. Columns reflect any DAEC/EAEC/ETEC/EIEC-containing pathotype. Mixed = ≥2 pathotypes co-assigned.

**Table 6:** Multivariable logistic regression results: DEC+ (any), DAEC, and EAEC.

Outcome/Predictor	OR	95% CI	*p*-Value	Interpretation
Outcome: DEC+ (any)—*n* = 102, events = 65, EPV = 21.7				
Age (per SD)	0.97	0.65–1.44	0.87	No significant age effect
Rainfall (per SD)	1.13	0.68–1.88	0.65	No significant rainfall effect
Male vs. Female	0.73	0.32–1.69	0.46	No significant sex difference
Outcome: DAEC (any)—*n* = 102, events = 37, EPV = 12.3				
Age (per SD)	0.74	0.47–1.15	0.17	Decreasing trend; not significant
Rainfall (per SD)	1.06	0.71–1.57	0.78	No rainfall effect
Male vs. Female	1.46	0.63–3.42	0.38	No significant sex difference
Outcome: EAEC (any)—*n* = 102, events = 16, EPV = 5.3 †				
Age (per SD)	0.48	0.21–1.08	0.07	Inverse trend (*p* = 0.07); underpowered
Rainfall (per SD)	1.11	0.70–1.75	0.65	No rainfall effect
Male vs. Female	2.52	0.72–8.81	0.15	No significant sex difference

OR, odds ratio; 95% CI, 95% confidence interval. All predictors standardised (mean = 0, SD = 1). EPV, events per variable. † EAEC model EPV = 5.3; interpret with caution. ns, not significant (*p* ≥ 0.05).

## Data Availability

The datasets generated and analysed during the current study, including per-sample pathotype calls, virulence family presence/GPMB matrices, and covariate data, are provided in Supplementary Tables S1–S3. Raw sequencing reads are deposited in the NCBI Sequence Read Archive (SRA) under BioProject accession PRJNA1484639.

## Supplementary Materials

The following supporting information can be downloaded at https://www.mdpi.com/article/10.3390/cimb48080794/s1.

## Abbreviations

A/E	Attaching-and-effacing
AAF	Aggregative adherence fimbriae
aEPEC	Atypical enteropathogenic *E. coli*
BH	Benjamini–Hochberg
DAEC	Diffusely adherent *E. coli*
DEC	Diarrheagenic *E. coli*
EAEC	Enteroaggregative *E. coli*
EIEC	Enteroinvasive *E. coli*
EPV	Events per variable
ETEC	Enterotoxigenic *E. coli*
EPEC	Enteropathogenic *E. coli*
FDR	False discovery rate
GPMB	Gene bases per million *E. coli*-aligned bases
IQR	Interquartile range
KMA	k-mer alignment
LEE	Locus of enterocyte effacement
LMICs	Low- and middle-income countries
LT	Heat-labile toxin
OR	Odds ratio
PCR	Polymerase chain reaction
SD	Standard deviation
ST	Heat-stable toxin
STEC	Shiga toxin-producing *E. coli*
SUP	Super-accuracy (basecalling model)
T3SS	Type III secretion system
tEPEC	Typical enteropathogenic *E. coli*
VF	Virulence family
